# Supplementary material for: Limited Health Risks in Performing Drug Reconstitution and Handling Tasks in Pharmacies—Results of an Occupational Risk Assessment Study
Source: J Occup Environ Med. 2023 Jan 16;65(4):e204–10. doi: 10.1097/JOM.0000000000002781 (PMC10090273; doi:10.1097/JOM.0000000000002781)
Supplement: SUPPLEMENTARY MATERIAL [file joem-65-e204-s001.docx]

**Limited health risks in performing drug reconstitution and handling tasks in pharmacies – results of an occupational risk assessment study**

**Running title:** Occupational exposure when handling drugs in pharmacies

**Key words**: drug reconstitution, drug handling, occupational exposure, inhalation exposure

**Word count:** 3634

**Abstract**

Objective: Some drugs need processing before they can be administered or dispensed.

We measured airborne exposure of pharmacy staff to small particles when performing these tasks.

Methods: Reconstitution of powdered drugs in vials; crushing, splitting, and counting of tablets; and opening of capsules, using different ventilation strategies, were investigated in 5 pharmacies following a worst case approach. Airborne particulate matter was determined for a range of particles sizes.

Results: Mean particle concentrations ranged from not-detectable to 1.03 µg/m^3^ (< 1 micrometer) and 589.7 µg/m^3^ (< 10 micrometer). Dust exhaust made tasks safer. Most hazardous was pouring out tablets from a bulk supply, and least hazardous was reconstitution of a powder for injection.

Conclusions: Occupational exposure during routine handling of drugs can occur, but the risks vary greatly with the nature and duration of the tasks.

Key words:

drug reconstitution, drug handling, occupational exposure, inhalation exposure

Learning Objectives:

After reading this article, the readers will be better able to:

- Understand and estimate the risks of exposure to medicinal products when performing handling and reconstitution tasks
- Understand the classification of medicinal products into risk classes
- Take appropriate protective measures for pharmacy staff when they perform the tasks that can lead to occupational exposure

**Introduction**

Pharmacies are work environments where a large number of different substances are handled, and where a large variety of work activities are performed. Assessing the risk of potential exposure of employees to hazardous substances, is therefore complex. Work should however, not pose a risk to the safety and health of workers. This basic occupational hygiene principle has been anchored in several binding European Directives.^1-3^ The actual risk for workers is a resultant of a) the inherent toxicity of handled substances and b) the potential exposure, stemming from the type of work that has to be executed. For example, an antibiotic drug such as doxycycline has a lower inherent toxicity than a classical cytotoxic such as cyclophosphamide, but crushing tablets of doxycycline in an open mortar to help a patient who has difficulty swallowing, is a riskier handling procedure than dispensing a commercial package with cyclophosphamide tablets in sealed strips. Hence, to ensure optimal worker safety, adequate risk analysis should be performed and, based on the outcomes of the analysis, adequate measures to mitigate the risks should be installed. Therefore, a common understanding of which substances pose a high inherent toxicity as well as which work procedures pose a high risk of exposure, is pivotal. With regard to the safe handling of drugs that have inherent carcinogenic, mutagenic and/or reprotoxic (CMR) properties, many studies have been performed, and both European regulations^1,3^ as well as guidelines stemming from authorities or professional organizations,^4,5^ are available. Research into the risks of handling of all other drugs however, is very scarce.

An early study from the Netherlands investigated occupational exposure in pharmacies when performing small scale production of medicinal products out of raw starting materials. Both dermal exposure as well as inhalation exposure was highly variable (4.7 µg to 166 mg and 0.13 µg/m3 to 2626 µg/m3, respectively) and depended on ventilation measures, physical form of the used materials (dry powder versus fluid) and the amount of substance used.^6^ Subsequently, a classification model for activities to assess inhalation exposure has been proposed^7^ but has not been evaluated in pharmacy environments. Moreover, this model investigated preparation of drugs out of raw materials^6^ which is distinctly different from handling tasks that are performed on commercially available drugs, where no open packages of pure active pharmaceutical ingredients are involved. Another study was performed in Japan, and investigated suspended particles of 25 different drugs in dust from 11 different pharmacy dispensaries. Both zopiclone as well as acetaminophen were detected in airborne particles in a total of 3 of the participating pharmacies.^8^

To our knowledge, no large studies evaluating exposure risk on handling tasks performed on commercially available drugs other than classical cytotoxic drugs, have been performed thus far. The aim of our study was to assess airborne exposure to medicinal products during routine handling tasks of commercially available drugs in the pharmacy. A pre- and post-intervention study was carried out, with the post-intervention based on installment of enhanced ventilation during the tasks that showed the highest exposure to particles in the pre-intervention measurements.

**Methods**

**Determination of tasks to be investigated**

The Royal Dutch Pharmacists Association (KNMP) installed a panel of pharmacists representing compounding pharmacies and non-compounding pharmacies and included both community and hospital pharmacists. The panelists were asked to compile a list of the handling activities that are performed routinely with commercially available drug products. Next, the panel was asked to determine a worst-case product for each of these handling activities, where exposure was deemed highest. For solid dosage forms, the worst case products were those with a relatively high level of active substance, brittle and uncoated in case of tablets, and dusty in case of powders. For fluid dosage forms, a low viscosity was chosen, as small droplet formation is more likely with a low than with a highly viscous solution. As to not unnecessarily expose the volunteers and researches during the study, relatively harmless drugs meeting the above requirements were then used to perform the experiments.

**Measurement protocol**

All measurements were performed in real-time. Exposure was measured continuously in the near field (situated in a hip-bag on the work bench next to the worker performing the tasks) and in the far field (situated >4 meters away from the worker) using Optical Particle Counter (OPC) sensors (OPC-N2, Alphasense, Braintree, United Kingdom). The OPCs measured particles ranging from 380 nm – 16 µm every second. Results were modified into mass concentrations assuming an average density of 1.6 g/cm^3^ and then stratified according to the moving average principle^9,10^, with averages given per minute for three categories: particulate matter PM 10 (particles with an aerodynamic diameter smaller than 10 micrometer); PM 2.5 (particles with an aerodynamic diameter smaller than 2.5 micrometer) and PM1 (particles with an aerodynamic diameter smaller than 1 micrometer). In addition to the OPC, quantitative near field measurements also used an Aerodynamic Particle Sizer (APS; 0.5-20 µm, TSI 3321, TSI Incorporated, Shoreview, USA) to measure particles every second. The use and influencing variables of these devices have been studied and reported previously.^11,12^ Measurements were performed in 3 community pharmacies, 1 hospital pharmacy and in the laboratory of the KNMP. Each task was performed by one or more pharmacy staff members who also perform these tasks as part of their daily work, and was repeated 1 to 4 times, to reach a total measurement time of approximately 20 -30 minutes. The duration of the tasks was timed by an independent researcher during this measurement period. The different participating pharmacies were assigned the different tasks based on which tasks they also perform on a regular basis in routine care.

**Assessment of exposure, risk analysis and intervention**

After the handling tasks were determined, an indicative pre-analysis was executed with the aim to identify which tasks lead to relatively high exposure for the worker (determined by comparing the near field exposures during performance of tasks and without performance of tasks). For this pre-analysis, measurements were performed with OPC and APS and each task was performed repeatedly by one member of the pharmacy staff for 20 to 30 minutes, or until sufficient data was collected. From this pre-analysis, the tasks that showed an increase in particle count were studied more in depth for quantitative results in two different pharmacies and executed by two different staff members.

Next, a risk analysis was performed based on the risk matrix that had previously been established by the Netherlands Association of Applied Scientific Research (TNO) and is used commonly in the Netherlands in compounding pharmacies.^6,13,14^ The basis for this risk matrix is the subdivision of pharmaceutical substances into 5 hazard categories, with category 1 being the lowest, and category 5 the highest. All commercially available active pharmaceutical ingredients and commercially available drug products are classified into one of the five categories in a national database, with the classification being a combined result of acute and chronic toxicity of the substance, derived from H-statements according to CLP/REACH regulations,^15^ available occupational exposure limits and toxicological and pharmacotherapeutic information. The classification system is shown in Appendix A, <http://links.lww.com/JOM/B251>. In addition, the exposure is stratified in the matrix, with also 5 distinct categories, ranging from very low to high, based on the geometric exposure of particles in the air. The combination of the intrinsic hazard of the substance with the exposure potential leads to risk classes visualized in the risk matrix (figure 1).

Risks were further investigated for three distinct work scenarios: a) a staff member performs handling tasks during a full work-day of 8 hours, corresponding to the situation in a hospital pharmacy where dedicated pharmacy technicians work in the compounding department, b) a staff member performs handling tasks during some but not all hours of the day, averaged on 2 hours, representing the situation where a pharmacy technician rotates over various tasks in the pharmacy and c) a staff member performs handing tasks only for a very short time of 15 minutes, representing the situation where a pharmacy technician is dispensing medication at the counter, and only once or twice a day, a prescription requiring a handling task is presented.

Finally, an intervention was performed for each handling task that resulted in a high exposure risk in the quantitative analysis (tasks were chosen based on visual inspection of the measurements, the tasks where a clear increase in exposure was visible, were chosen for the intervention). This intervention consisted of increasing ventilation measures by performing the tasks in a dust exhaust cabinet (air class 5 according to ISO 14644-1, with air flow velocity of 0,25-0,50 m/s) with the aim to reduce the risk classification. After the intervention, the measurements were performed again to evaluate the effectiveness of the intervention, using the same protocol.

**Results**

**Determination of tasks to be investigated**

Two community pharmacists, two hospital pharmacists and two staff members of the KNMP each individually compiled a list of handling tasks that are performed routinely. The lists were collated and compared, after which consensus was established that the following tasks are most likely to potentially lead to exposure:

1. Reconstitution of a powder for injection on a workbench, using needles
2. Reconstitution of a powder for injection in a safety cabinet, using needles
3. Manually deblistering tablets or capsules from strips
4. Crushing tablets in an open mortar on a workbench
5. Splitting tablets without a score line on a workbench with a splitter
6. Splitting tablets with a score line on a workbench by hand
7. Reconstitution of a powder for oral suspension or solution on a workbench
8. Opening capsules and pouring out the contents on a workbench
9. Repacking of tablets from a bulk supply into small packages by hand on a workbench

For the parenteral reconstitution, the use of needles was chosen over the use of spikes or close-system transfer devices (CSTDs), as the use of needles was thought to be the worst case in this respect, since the use of needles can form an open connection with the surroundings, and a positive pressure in the vial during reconstitution, can enhance the potential of aerosol formation when using a needle.

Next, pharmaceutical products were chosen to act as a worst case when looking at exposure, without compromising the safety of the staff members who were to perform the experiments. For the reconstitution of powder for injection or oral administration (tasks 1, 2 and 7), amoxicillin was chosen because this represents a product with a large volume of active ingredient. The reconstitution solvent chosen was water for injection, as this represents a low viscosity fluid. For the tasks involving tablets (tasks 3,4,5,6 and 9), paracetamol and vitamin C were chosen, based on their properties of being relatively brittle and non-coated tablets that can easily give of particles. For the opening of capsules (task 8) large size talcum capsules were chosen, because talcum is a substance with rheology properties making it prone to dusting.

**Indicative measurements to determine the worst case(s)**

For the indicative measurements, all nine tasks were performed according to a fixed protocol in September of 2017 (appendix B, <http://links.lww.com/JOM/B252>). Tasks 1, 4 and 8 were performed in a hospital pharmacy, tasks 3, 5, 6, 7 and 9 in a community pharmacy, and tasks 2 and 4 in the laboratory of the KNMP. Each pharmacy was assigned a code (A through C). The results from the indicative measurements are shown in figure 2. As can be seen, tasks 4, 7, 8 and 9 result in distinct elevation of particles in the near field next to the staff member, when compared to the far field measurements. Therefore, these tasks were chosen to be measured more in depth in the second part of the study, in two other community pharmacies (pharmacy D and E).

**Quantitative analyses**

For the quantitative analyses, particle counts from the first part of the study were combined with results from additional measurements of tasks 4, 7, 8 and 9, which were done in two additional separate pharmacies during December 2017 using the APS results. Task 9: Repacking of tablets from a bulk supply into small packages by hand, was found to be performed by workers in two distinct ways: a) pouring tablets over from a large into a small package, or b) counting them out using a tablet counter (see appendix B for more details, <http://links.lww.com/JOM/B252>). Thus, task 9 was subdivided in 9a and 9b and both methods were measured. Results, stratified to particle size, showed a large variability (table 1), with the highest particle counts in the task “repacking of tablets from a bulk supply into small packages by hand when pouring the tablets from one package into another” (task 9a: maximum 589.65 µg/m^3^ for PM10), followed by splitting tablets without a score line on a workbench with a splitter (task 5: this task was measured in only one pharmacy, hence no maximum between locations can be determined) and opening capsules and pouring out the contents on a work bench (task 8: maximum 43.14 µg/m^3^ for PM10). When comparing task 9a and 9b, the use of a tablet counter reduced the number of particles almost fifty-fold. The tasks that resulted in no particle exposures on average were the tasks involving handling of parenteral drugs: reconstitution from a vial. Here, the number of particles was not raised when compared to the far field measurements. The indicative first part of the trial did not show a clear risk for task 5, which might in hindsight be a result of a short pause between performing handling task 9 and then task 5 (figure 2) – and the relatively high background particles still present from performing task 9. As not only the mean or standard deviation is relevant, very short peak exposures could be relevant as well, boxplots of the four worst case task measurements including each of the three different particle counters were made (figure 3). Also here, it is clearly visible that task 9a gives the highest particle burden with a clear difference between the near field and the far field. Moreover, the two different particle counters that were used, APS and OPC, give very comparable results. Finally, the variability and maximum particle counts show the greatest range in task 7 and task 9b.

**Risk analysis, risk mitigation and intervention**

After the quantitative measurements, a risk analysis was performed based on the risk matrix (figure 1). For this risk analysis, the measured concentrations of PM 10 were used (because PM 1 and PM 2.5 are part of PM 10, and because PM 10 is inhalable). A risk analysis was done for three scenario’s (8-hour work shift, 2 hour rotation on handling practices, 15 minutes of handling practices). A high risk was found for drugs of hazard class 4 and 5 for tasks 3 through 9 for the 8-hour work shift as well as for the 2 hour rotation scenario. Moderate risks were found for drugs of hazard class 3 for tasks 4, 5, 8 and 9a (pouring out tablets) in the 8-hour work shift and task 9a for the 2 hour rotation scenario. In addition, drugs of hazard class 2 also showed a moderate risk in the 8-hour work shift for task 9a.

When looking at the scenario of just 15 minutes of performing handling tasks per day, drugs of hazard class 4 and 5 resulted in high risks when performing tasks 4, 5, 8 and 9a. For the tasks 1 and 2, the reconstitution tasks, only low risks were found independent of the hazard class of the drugs. This means that for regular reconstitution tasks, no safety cabinet or other ventilation measure is required for the protection of the workers. Of course, following the (inter)national guidelines on safe handling of carcinogenic and or mutagenic substances, reconstitution of those drugs is done with extra technical safety measures.

The four tasks that showed the highest variability in measurements were: splitting tablets without a score line on a workbench with a splitter (task 5); splitting tablets with a score line on a workbench by hand (task 6); reconstitution of a powder for oral suspension or solution on a work bench (task 7); opening capsules and pouring out the contents on a work bench (task 8). The ninth task showed a high risk of exposure when using the pouring method, but this was reduced to a lower risk classification when using the counting out protocol with the tablet counter. For the four tasks with the highest variability in exposure, a dust exhaust was deemed a good intervention to reduce exposure. To investigate the effect of this risk mitigation measure, these four tasks were performed and measured again in March of 2018 following the exact same measurement protocol (appendix B, <http://links.lww.com/JOM/B252>) but in duplicate: with and without dust exhaust. For task 8, only with dust exhaust was measured, as there were already duplicate data from the first part of the study without dust exhaust. The results are given in Table 2. As can be seen, the particle concentrations decrease for each task for each particle size category. The effectivity of the use of a dust exhaust, given as the percentage decrease in particles for each task for each particle size category, ranged from 57% to 94%.

After the measurements with the dust exhaust on, the risk analysis was performed again. These results are visualized in figure 4. When performing handling tasks for 15 minutes, only task 9a with drugs from hazard classes 4 and 5 pose a high risk. Therefore, we recommend not using this procedure, but always using the method of counting out with a tablet counter. When performing handling tasks for 2 hours, also task 8: opening capsules and pouring out the contents on a work bench, gives a high risk of exposure when drugs of hazard classes 4 and 5 are handled. Hence, in these cases, a dust exhaust is not enough. We recommend additional measures, either performing these tasks in a safety cabinet or using a respiratory mask of filtering face piece (FFP) class 2 or higher. When workers are performing handling tasks as a full time job (8-hour work shifts) tasks 3, 4, 7, 8 and 9 with drugs of hazard class 4 and 5 result in a high risk, even when working with a dust exhaust. Hence, also in these cases, additional protective measures are required.

**Discussion**

To our knowledge, this is the first multicenter study into occupational exposure when performing handling tasks in pharmacies that incorporates handling of all medicinal products. We have shown shows that for drugs of hazard class 1 through 3, very limited health risks occur when performing routine tasks. For workers who perform handling tasks for 2 or 8 hours per day, a dust exhaust should be used for all the tasks involving non-aseptic handling, and additional protection in the form of a face mask with a ventilation valve is mostly required when drugs of hazard class 4 or 5 are being handled. For reconstitution tasks (dissolving a powder for injection or infusion with a needle in a vial) no additional protective measures are necessary.

Although many studies have been performed on handling of high hazard drugs (so called CMR substances), very little attention has been given to handling of non-CMR drugs. One previous study looked at classical small scale preparation of drugs in pharmacies, investigating preparation of a dosage form out of raw materials.^6^ This is distinctly different from the handling tasks described in our study. When comparing our outcomes to previously published studies, we confirm the data from Japan, where a beneficial effect of dust exhausts was described.^8^ Our results are not confirming one previous study on the reconstitution of antibiotics that promote the use of CSTDs to do so.^16^ In fact, using a needle is adequate in that case and even the use of a safety cabinet or isolator is not required. However, the latter may of course be useful when a drug that is reconstituted is not administered immediately, to be able to guarantee a good microbiological safety of the drug. Using a needle in general (drugs of hazard class 1-4) or a spike for CMR substances (drugs of mostly hazard class 5) is sufficient from an occupational perspective, which is in line with a previous Cochrane review.^17^ For CMR substances, to enable containment of possible contamination stemming for example from the outside of cytotoxic drug vials, the use of a safety cabinet or isolator is also recommended.^5^ Although our study was done in pharmacies, the results of the reconstitution of intravenous drugs are also applicable to nurses who perform such tasks in clinical wards, as the handling steps are the same.

Some limitations of the present study that should be taken into account are the fact that we used model drugs, based on a worst case scenario, and that we had a single nation design. In addition, we measured only inhalation, and not dermal exposure. Since all tasks described and studied should be done wearing gloves in the Netherlands, we assume that inhalation is the most important exposure route. However, future research including measurement of dermal exposure or investigating tasks that are performed less frequently, are still warranted. Also, potential exposure to drug residues of cleaning personnel in pharmacies has not been investigated yet. In that perspective, the potential hazard of all other personnel not directly involved in the handling tasks, warrants further investigation, for example by determining the nature of particles found in the far field measurements. Finally, the development of marketed pharmaceutical products with better occupational safety properties (e.g. non-brittle tablets, better score lines on tablets, enclosing powders for oral solutions) is an area that would be of great interest and potential benefit for personnel working on a daily basis with these products.

In conclusion, the present study demonstrates that health risks are limited when tasks involving handling of medicinal products are performed in pharmacies for short periods of time.

References

1. European Parliament and the Council of the European Union. Directive 92/85/EEC of the European Parliament and of the Council on the introduction of measures to encourage improvements in the safety and health at work of pregnant workers and workers who have recently given birth or are breastfeeding (tenth individual Directive within the meaning of Article 16 (1) of Directive 89/391/EEC). Available at: <https://eur-lex.europa.eu/legal-content/EN/TXT/?uri=celex%3A31992L0085> Accessed august 28, 2017.
2. European Parliament and the Council of the European Union, April 7^th^, 1998. Directive 98/24/EC on the protection of the health and safety of workers from the risks related to chemical agents at work (fourteenth individual Directive within the meaning of Article 16(1) of Directive 89/391/EEC). Available at: https://eur-lex.europa.eu/legal-content/EN/TXT/?uri=CELEX%3A31998L0024 Accessed august 28, 2017.
3. European Parliament and the Council of the European Union. Directive 2004/37/EC of the European Parliament and of the Council on the Protection of Workers from the Risks Related to Exposure to Carcinogens or Mutagens at Work (Sixth Individual Directive within the Meaning of Article 16[1] of Council Directive 89/391/EEC). Official Journal of the European Union April 29, 2004; L 158: 50–76. Available at: www.reach-compliance.eu/english/REACH-E/engine/sources/regulations/2004-37-EC.pdf Accessed august 28, 2017.
4. NIOSH Preventing occupational exposure to antineoplastic and other hazardous drugs in health care settings. 2004; Publication Number 2004-165.
5. European Society of Oncology Pharmacy (ESOP) Quapos: Quality Standard for the Oncology Pharmacy Service. 6^th^ edition 2018. Available at: <https://esop.li/quapos/> Accessed September 30, 2021.
6. Tielemans E, le Feber M, Groenewold M, et al. (2006) Risico’s tijdens het bereiden van geneesmiddelen in de apotheek (Dutch). TNO rapport V6288. Available at: <http://www.rifas.nl/documentatie/rp_tno_v6288-2.pdf>. Accessed November 30, 2021.
7. Marquart H, Schneider T, Goede H, et al. Classification of occupational activities for assessment of inhalation exposure. *Ann Occup Hyg* 2011;55:989-1005.
8. Inaba R, Hioki A, Kondo Y, Nakamura H, Nakamura M. Suspended particle and drug ingredient concentrations in hospital dispensaries and implications for pharmacists’ working environments. *Environ Health Prev Med* 2016;21:105-110.
9. Pitz M, Schmid O, Heinrich J, et al. Seasonal and diurnal variation of PM2.5 apparent particle density in urban air in Augsburg, Germany. *Environ Sci Technol* 2008;42:5087-5093.
10. Pitz M, Cyrys J, Karg E, Wiedensohler A, Wichmann HE, Heinrich J. Variability of apparent particle density of an urban aerosol. *Environ Sci Technol* 2003;37:4336-4342.
11. Ruiter S, Kuijpers E, Saunders J, et al. Exploring Evaluation Variables for Low-Cost Particulate Matter Monitors to Assess Occupational Exposure. Int J Environ Res Public Health 2020;17:8602.
12. Goede H, Kuijpers E, Krone T, et al. Future Prospects of Occupational Exposure Modelling of Substances in the Context of Time-Resolved Sensor Data. *Ann Work Expo Health* 2021;65:246-254.
13. Bouwman-Boer Y, Ng S, Crauste-Manciet S. (2016) Chapter 26 Occupational Safety and Health. In: Bouwman-Boer Y, Fenton-May V, Le Brun P ed. Practical Pharmaceutics, the Hague, the Netherlands. ISBN  9783319158136
14. KNMP - Royal Dutch Pharmacy Association, Risico Instrument Farmaceutische Stoffen (Dutch). Available at: [www.rifas.nl](http://www.rifas.nl) Accessed December 14, 2021.
15. European Parliament and the Council of the European Union. Directive 2008/1272/EC of the European Parliament and of the Council Classification, labelling and packaging of substances and mixtures. Available at <https://eur-lex.europa.eu/LexUriServ/LexUriServ.do?uri=OJ:L:2008:353:0001:1355:en:PDF> Accessed August 28, 2017.
16. Sessink PJM, Nyulasi T, Haraldsson ELM, Rebic B. Reduction of Contamination with Antibiotics on Surfaces and in Environmental Air in Three European Hospitals Following Implementation of a Closed-System Drug Transfer Device. *Ann Work Expo Health* 2019;63:459-467.
17. Gurusamy KS, Best LM, Tanguay C, Lennan E, Korva M, Bussières JF. Closed-system drug-transfer devices plus safe handling of hazardous drugs versus safe handling alone for reducing exposure to infusional hazardous drugs in healthcare staff. *Cochrane Database Syst Rev* 2018; DOI: 10.1002/14651858.CD012860.pub2.

Figure 1 Risk matrix for inhalation risks during compounding and reconstitution in the pharmacy. Risks are stratified into three classes: small (green), moderate (blue) and high (red). GM = geometric mean, exposure is given as particle exposure in air.^14^

Figure 2 Moving average of OPC measurements during the experiments over time. Green lines represent particles with a particle size <1 micrometer, purple lines represent particles with a particle size <2.5 micrometer and yellow lines represent particles with a particle size <10 micrometer. Top: measurements in pharmacy A, Middle: measurements in pharmacy C, Bottom: measurements in pharmacy B

Figure 3: Boxplots of the measured mass concentration (in µg/m^3^) on a logscale of the APS (red) OPC in near field (green) and OPC in far field (blue) for each of the three particle size groups (PM1 = A, PM 2.5 = B and PM 10 = C). Results are corrected for background. FF = far field, NF = near field

Figure 4 Risk levels of the handling tasks with and without ventilation measures for 15 minutes, 2 hours and 8 hours of performing these handling tasks
